# Maintenance of atrial fibrillation by reciprocal triggering of competitive focal sources: a hypothesis

**DOI:** 10.3389/fcvm.2026.1910201

**Published:** 2026-10-05

**Authors:** Alexander Burashnikov

**Affiliations:** Lankenau Institute for Medical Research, Wynnewood, PA, United States

**Keywords:** atrial fibrillation, delayed afterdepolarization activity, focal source, reentry, spiral wave

## Abstract

The primary mechanism of atrial fibrillation (AF) maintenance remains debatable. However, contemporary atrial surface AF mapping studies readily detect multifocal activity, less readily “incomplete reentries” (defining them as reentries or false reentries) and never or rarely full-circuit reentries. Most of the surface multifocal waves during AF are short-lasting (1–3 waves) and they appear to be largely breakthroughs. Some of the multifocal activities during AF, particularly repetitive/sustained, can be explained with reciprocal triggering of delayed afterdepolarization (DAD) activities. Indeed, the condition that causes DAD activity (i.e., intracellular calcium abnormalities plus rapid activation) is continuously present during AF throughout the atria, that may provide a sufficient “arrhythmogenic reserve” for the generation of multiple competitive focal sources sustaining AF. In such scenario, there should be commonly one dominant source at a time along with subsidiary active and/or latent/overridden sources (the currently fastest sustained source acts to override all the others, covering all, most, or a large part of the atrium at a time). This may account for a spatiotemporal periodicity usually recorded during AF. Sustained focal sources should self-terminate/decelerate sooner or later and/or be overridden by currently faster sources, entailing a periodical switching of dominant sources (the currently “fittest” dominates). Anti-AF success of the Cox-Maze and pulmonary vein isolation procedures, separating the main atrial arrhythmogenic regions, can be generally explained with the interruption of mutual triggering of multiple DAD sources. Thus, reciprocal triggering of competitive DAD sources may broadly account for the maintenance and spatiotemporal periodicity of AF as well as for the anti-AF success of the Cox-Maze and pulmonary vein isolation procedures.

## Introduction

The primary mechanism driving atrial fibrillation (AF) is disputable currently, with the prevailing dogma being “reentry drives AF” (1–4). Contemporary AF mapping studies, however, readily record focal activation patterns (5–15), either without reentries at all (5–8, 16) or, with few exceptions (17–20), more likely than reentries (i.e., “incomplete reentries” defined as reentries) (10–12, 14, 21–26). Importantly, focal waves can reflect real focal sources and breakthroughs (7, 8, 27), and the reported reentries can be real reentries and false reentries (28–32). To resolve these issues a precise 3-D mapping technology allowing the precise determination of AF mechanisms, is required, but not available presently (2, 33). There is a substantial spatiotemporal periodicity during AF (21, 34–41), pointing to a stationary source(s) sustaining AF and arguing against random sources driving AF (21, 40, 41). The Cox-Maze procedure (CMP) is the most effective approach against AF, and the original rationale behind the CMP is that “AF is sustained by multiple simultaneous reentries” and, therefore, atrial segmentation reduces the chance of current reentries to continue and novel reentries to appear (42, 43). The pulmonary vein isolation (PVI) is a proven anti-AF strategy and the key explanation for its success is that “PV regions generate the triggers that induce reentries sustaining AF” and, therefore, the PVI is effective against AF (4, 44, 45). These rationales for the CMP and PVI may be questioned by the results of contemporary AF mapping studies detecting either no reentry at all or a single reentry while regularly finding focal activations (5–12, 14, 15, 24, 46). Also, numerous approaches targeting reentry substates (i.e., fibrosis, Complex Fractionated Atrial Electrogram [CFAE], Focal Impulse and Rotor Modulation [FIRM], etc.) generally failed to reduce AF occurrence vs. PVI alone in the randomized controlled trials (RCT) (47–53). This data does not appear to support the “reentry drives AF” dogma. The current analysis reviews AF mapping data and attempts to correlate the properties of focal arrhythmic sources with 1) multifocal appearance of atrial activations during AF, 2) spatiotemporal periodicity during AF, and 3) anti-AF success of the CMP and PVI.

### Limitations of AF mapping technologies

There are important limitations in the available AF mapping technologies, usually not allowing the precise determination of AF maintenance mechanism(s) (33). These limitations include 1) restricted atrial mapping area, 2) commonly insufficient resolution, 3) the absence or partial information on the intramural activation, 4) uncertainties in the interpretation of signals/electrograms, 5) short duration of uninterrupted mapping time (usually ≤ 30 s), etc (1, 33). There is a complex 3-D propagation during AF (33), indicating that intramural/transmural propagation may be involved in the maintenance of AF. However, a mapping technology permitting the precise following of the activation propagation in 3-D has not been developed yet (2, 33). There are novel promising mapping approaches [such as forward-solution mapping (54)] aiming to overcome the problems of the currently available AF mapping methods. The validity of these novel approaches is yet to be determined. Notably, the phase mapping method, introduced by Grey et al. (39) in 1998 was aimed to improve “conventional” approaches, and this method was developed following the failure of finding reentry during vagally-mediated AF (that was “known” to be reentrant) using “conventional” approach by this group (55). Later, the phase mapping was found to often (28), or even always (31, 32, 56), detect false reentries. It is important to scrutinize novel AF mapping approaches to prevent the appearance of another method commonly or always finding non-existing reentries.

### Circular activations during AF

It has been generally agreed for decades that reentry is the primary driver for AF (2, 57–59). Yet, it is increasingly recognized that solid evidence for reentry as the primary driver of AF is absent (1–4). It was accepted in ∼ 1980–1990th that the dominant mechanism of AF maintenance was simultaneous multiple reentries (this acceptance was essentially based on the atrial surface mapping studies) (42, 57, 59, 60). With the development of AF mapping technologies, however, simultaneous multiple reentries during AF have not been detected on the atrial surfaces (5, 7–11, 20, 27, 61–66). Moreover, increasingly more AF mapping studies do not detect reentry at all (5–7, 16, 61, 62, 65). Most of those who readily find reentries during AF use a low-density mapping and/or the phase mapping method (17–19, 67–72). These approaches often (28–30), if not always (31, 32, 56), detect false reentries. Also, practically all reported reentries are “incomplete reentries” defined as reentries (17, 18, 68–70, 73–77), i.e., they can be true and false reentries (7, 28–32). “Reentries” can be recorded even at the absence of AF during rapid atrial pacing (78).

Contemporary AF mapping studies using a high-density atrial surface mapping and “conventional” interpretations of electrograms either do not detect reentries at all (5, 7, 8, 31, 32, 61, 62, 65) or record reentries only rarely, one at a time, and short-lasting (usually < 1 s) (10, 28, 76, 79). So, the available data indicate that “surface” reentries are unlikely to be the primary driver of AF. However, the absence or rare presence of reentries on the atrial surfaces during AF does not exclude their existence in the atrium, considering the limitation of the currently available AF mapping technologies (described above). Particularly, reentries may not be detected on the atrial surfaces because they may occur intramurally or transmurally as well as because only part of the reentrant circuit may run on the atrial surface, with the rest of it running intramurally.

Very limited data exists regarding transmural and intramural reentries. Using simultaneous endocardial and epicardial mapping during AF, some groups reported transmural reentries (71, 80), but many others, using a high-density mapping, did not find them at all (27, 65, 66). Why? Hansen et al. (20, 81), using simultaneous sub-epicardial and sub-endocardial optical mapping of isolated human right atrial preparations (with a 1–4 mm “deepness vision”) consistently recorded stable intramural anatomical reentries during AF (lasting ≥ 10 min), predominantly in sub-endocardium. In another study, Hansen et al. (81) readily detected such stable intramural subendocardial reentries on the endocardial surface as well, using a low-density multielectrode endocardial catheter (64-electordes, 9 × 9 mm^2^ resolution) and the phase mapping. According to these data (20, 81), most/many of the intramural reentries during AF should be detected on the atrial endocardial surface. Hansen et al. study (81) essentially confirmed Narayan et al. (17) and Miller et al. (68) clinical data, regularly founding *stable* reentries on the endocardial surface during AF using a low-density multielectrode endocardial catheter and phase mapping. However, such “stable endocardial/intramural reentry” data (17, 68, 81) have not been reproduced by numerous AF endocardial mapping studies, either not finding reentries at all or detecting them only short-lasting (<1 s) (10, 12, 15, 16, 27, 64–66, 82). So, the occurrence of transmural and intramural reentries during AF is poorly studied and controversial. A reliable 3-D mapping technology, unavailable presently (2, 33), is needed for the detection of transmural/intramural AF.

The development of reentry is commonly based on reentrant substrate (largely electrical and structural remodeling). There have been many anti-AF ablation approaches targeting reentry substrates (i.e., fibrosis, CFAE, FIRM, various linear lesions, etc.), and these approaches have failed to reduce AF occurrence vs. PVI alone in the well-controlled RCTs (47–52). These negative results do not refute the “reentry drives AF” dogma, considering the complexities of ablation procedures, uncertainties in finding AF sources in the patients, etc., but clearly do not support it.

The dominant role of reentry in AF maintenance was thought to be proven 1–3 decades ago, and it was based on atrial surface mapping studies. However, with improvement of AF mapping technologies, reentries on the atrial surface during AF have not been detected consistently, with increasingly more high-density multielectrode studies not detecting “surface” reentries at all. Data on the presence of intramural/transmural reentries during AF is scarce and controversial. The dominant role of reentry in AF maintenance is now subject to debate.

### Focal activations during AF

In contrast to reentries, practically all AF mapping studies readily detect focal activation pattens on the atrial surfaces (5–15, 31, 63, 83–85). Moreover, increasingly more AF mapping studies record either focal waves without reentry or predominantly focal activity (5–12, 14, 15, 23, 26, 61, 62). Multiple focal waves are regularly detected during AF (6–8, 13). Individual focal waves are most commonly single events (8, 27, 65, 66) but focal waves can be consecutive activations as well, ranging from short-lasting (< 1 s) (8, 76) to sustained (> 30 s) (6, 7, 12, 14, 86). The exact sustainability of these sustained focal waves has not been determined due to relatively brief mapping recording periods (usually lasting 5–32 s) (6, 7, 12, 14, 15). Intermittent focal activations during AF (lasting >1 - < 30 s) can occur and re-occur in the same atrial locations (6, 7, 23, 86). When considering only repetitive focal waves recorded on the atrial surfaces during AF, some mapping studies often detected sustained and intermittent focal activations (6, 7, 14, 86), while many others recorded only short runs of focal waves (most commonly < 1 s) (5, 62, 65, 76). The reason for this discrepancy is not clear. The size of atrial surface mapping may be an important factor. Lee et al. (6, 7), using bi-atrial mapping (510–512 electrodes; covering most of the atrial epicardial surface in both atria), often detected simultaneous sustained and intermittent focal sources. Those who readily found short-lasting but never sustained focal waves mapped a relatively small atrial surface area (< 10 cm^2^; 128–244 electrodes) (5, 62, 65, 76). Yet, sustained focal waves could be detected with a relatively small atrial mapping area as well (14, 15, 86).

It is generally accepted that some of the focal waves on the atrial surface are directly caused by true focal sources and these sources, particularly repetitive/sustained, may drive AF or contribute to AF maintenance (7, 10, 15, 27, 31, 87). Yet, many/most of the surface focal waves, particularly short-lasting, are breakthroughs (7, 8, 27). Breakthroughs on the atrial surface during AF can be induced by transmural/intramural reentries (20, 71, 80, 81) and wavelets (“permanently” wandering or originating from a remote stable source) (8, 27, 78). The occurrence of intramural/transmural reentries during AF by itself is controversial and poorly studied (as discussed above), putting aside the incidence of focal breakthroughs induced by such reentries. Indeed, many studies utilizing a high-density simultaneous epicardial and endocardial atrial mapping and readily recording focal waves during AF did not find evidence that any of these focal waves were caused by reentries (in fact, transmural reentries were not even detected) (8, 27, 65, 66).

Most, if not all, of the focal breakthroughs are short-lasting, and they are likely to be caused by transmural wavelets. Based on high-density simultaneous epicardial and endocardial atrial mapping data, it was deduced that short-lasting focal surface waves during AF were largely (in 65%–86% cases) caused by transmural wavelets and most of the rest focal waves were induced by ectopic impulses but not by reentries (in these studies vast majority of focal waves lasted < 1 s) (8, 27, 65, 66). The origin of wavelets causing focal breakthroughs on the atrial surface during AF is poorly defined. Where do they start? If these wavelets are not induced by remote rapid sources, they must originate from the last beat(s) preceding the start of AF, “permanently” wandering in the atrium during AF for hours/days/weeks/months (spatiotemporally changing due to wave-breaks, conduction blocks, etc.). Is it realistic? It was proposed that randomly traveling wavelets themselves may maintain AF (8, 88). However, repetitive atrial activations are commonly recorded during AF (21, 34–41, 89, 90) and they can be readily explained with stationary driver(s), but not with random sources. Note also that fibrillatory wavelets and focal breakthroughs can be readily explained with a stable remote source. Indeed, fibrillatory wavelets in the atrium are naturally induced by a single stable rapid source (58, 78), and focal breakthroughs on the atrial surface can be caused by a remote rapid atrial pacing (i.e., by “focal source”) at the absence of AF (78). Moreover, focal breakthroughs can emerge even during sinus rhythm, provided that there is a significant epi-endo electrical dissociation (91). Most of the short-lasting focal wavers appear to be breakthroughs caused by fibrillatory wavelets coming from stable remote sources (of unknown mechanism). Some of the atrial surface focal surface activations (particularly sustained) recorded during AF seem to be directly caused by focal sources.

Thus, focal waves are readily recorded in practically all contemporary AF mapping studies. Focal sources may be the primary driver of AF.

### Focal mechanism for AF maintenance: DAD activity?

The nature of focal activations recorded during AF is difficult to determine (focal waves can be caused directly by real focal sources as well as by all sorts of breakthroughs, as discussed above), putting aside specific arrhythmic mechanisms behind the focal sources (1, 3, 92). Therefore, practically all available data on specific focal mechanisms have been obtained in isolated tissue preparations and single cells (93–95). These data *in vitro*, however, do not well reproduce rapid atrial focal sources *in vivo* (3). Principally, while there are focal AF and atrial tachycardias (7, 13, 31, 63, 83–86, 96), the rate of focal activities recorded in atrial isolated tissue preparations and single cells is too slow to account for the rate of AF and even atrial tachycardia (95, 97–102). It appears that very rapid atrial focal source activity can take place only in natural or close to natural setting (i.e., *in vivo*, *in situ*, and in coronary-perfused hearts/atria) but not in superfused atrial tissues or isolated single cells (3, 92). Yet, focal source data obtained in isolated atrial tissue and single cells are still the best available for the deduction of focal mechanism(s) capable of sustaining AF.

There are generally two types of focal activity, i.e., automaticity (normal and abnormal) and triggered activity (early and delayed afterdepolarization; EAD and DAD, respectively) (94). Normal automaticity is generally too slow to maintain AF. Classical abnormal automaticity develops at a depolarized resting membrane potential (RMP) (93, 94). The RMP of focal drivers of AF is unknown. Yet, available data indicate that AF acts to hyperpolarize atrial RMP [secondary to a reduced I_K1_ (103–105)], arguing against classical abnormal automaticity as a driver of AF. Classical EAD activity develops in the setting of prolonged repolarization and bradycardia, while AF maintenance is associated with shortening of atrial repolarization and rapid rates (94). Therefore, classical EAD activity is very unlikely to contribute to AF maintenance. Yet, late phase 3 EAD activity (a mixture of EAD and DAD), occurring in the setting of rapid activation rates, short atrial repolarization, pauses, and intracellular calcium loading (106, 107), hypothetically, may contribute to AF maintenance.

DAD activity develops in the setting of intracellular calcium (Ca_i_) abnormalities and is triggered by rapid activation (Figure 1) (94, 95, 108). Both these factors are continuously present during AF throughout the atria and, conceivably, may provide a sufficient multifocal “arrhythmogenic reserve” for the maintenance of AF. So, among the known focal arrhythmic mechanisms (93, 94), DAD activity is most closely suitable for the explanation of the multifocal sources recorded during AF*.* It appears that mutual triggering of multiple DAD sources may maintain AF (Figure 2). The involvement of DAD activity in AF maintenance has been proposed in many studies (9, 108–113). Persistent/chronic AF enhances sarcoplasmic calcium (SR) leak that promotes DAD activity in atrial cells (109, 110, 112). Interestingly, atrial stretch (a typical feature in AF patients) may induce diastolic SR calcium leak (114). It appears that both the rapid activation rate and stretch additively or synergistically cause SR leak during AF promoting DAD activity.

**Figure 1:**
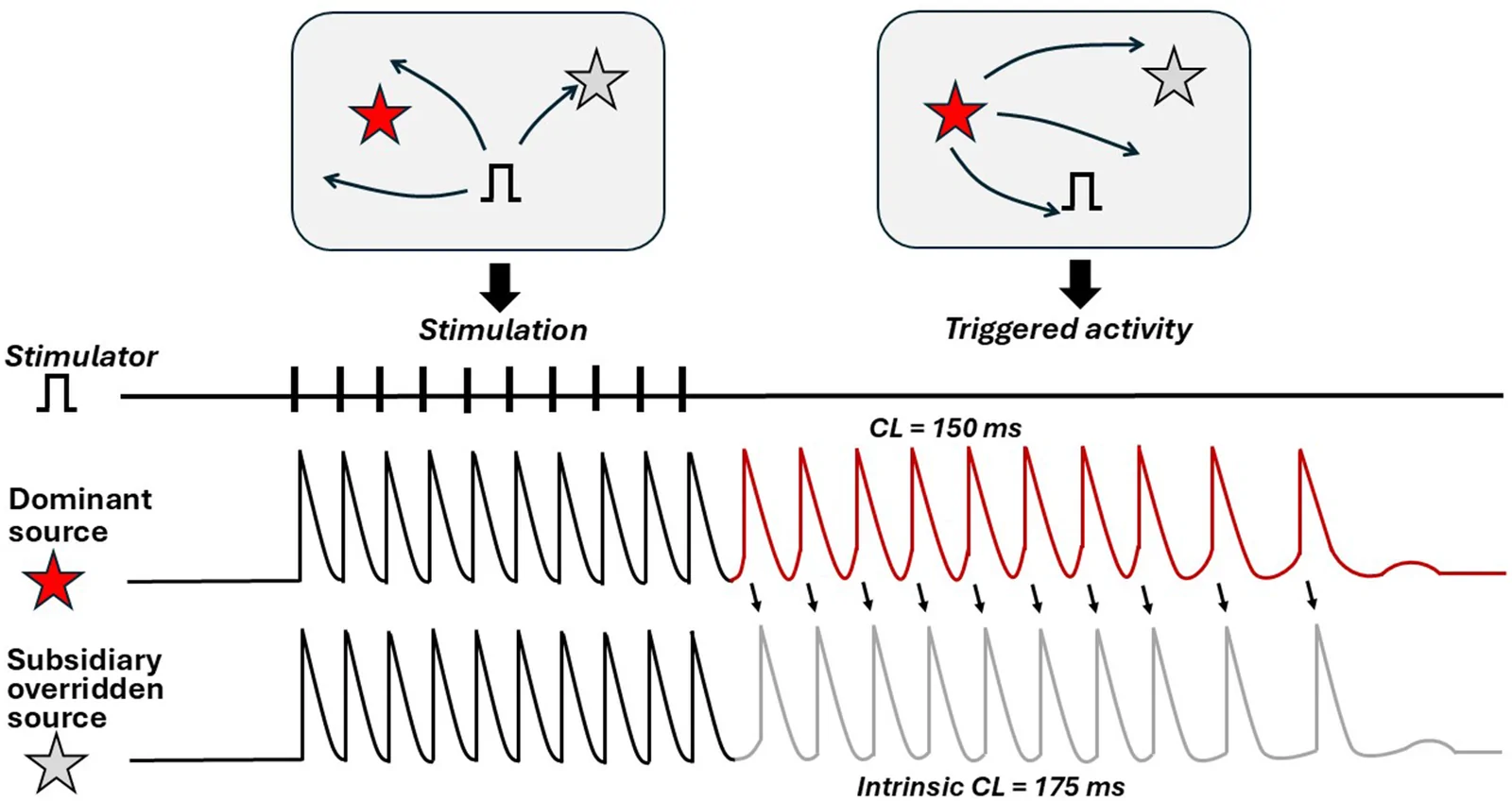
Induction of DAD activity in an isolated cardiac tissue slice: a simplistic schema. In the presence of elevated intracellular calcium, rapid pacing readily induces DAD activity. There are multiple potential sources of DAD activity in the tissue, and the fastest source takes the lead, overriding all the other DAD sources (“the fittest dominates”). DAD activity usually spontaneously terminates with slowing the firing rate.

**Figure 2:**
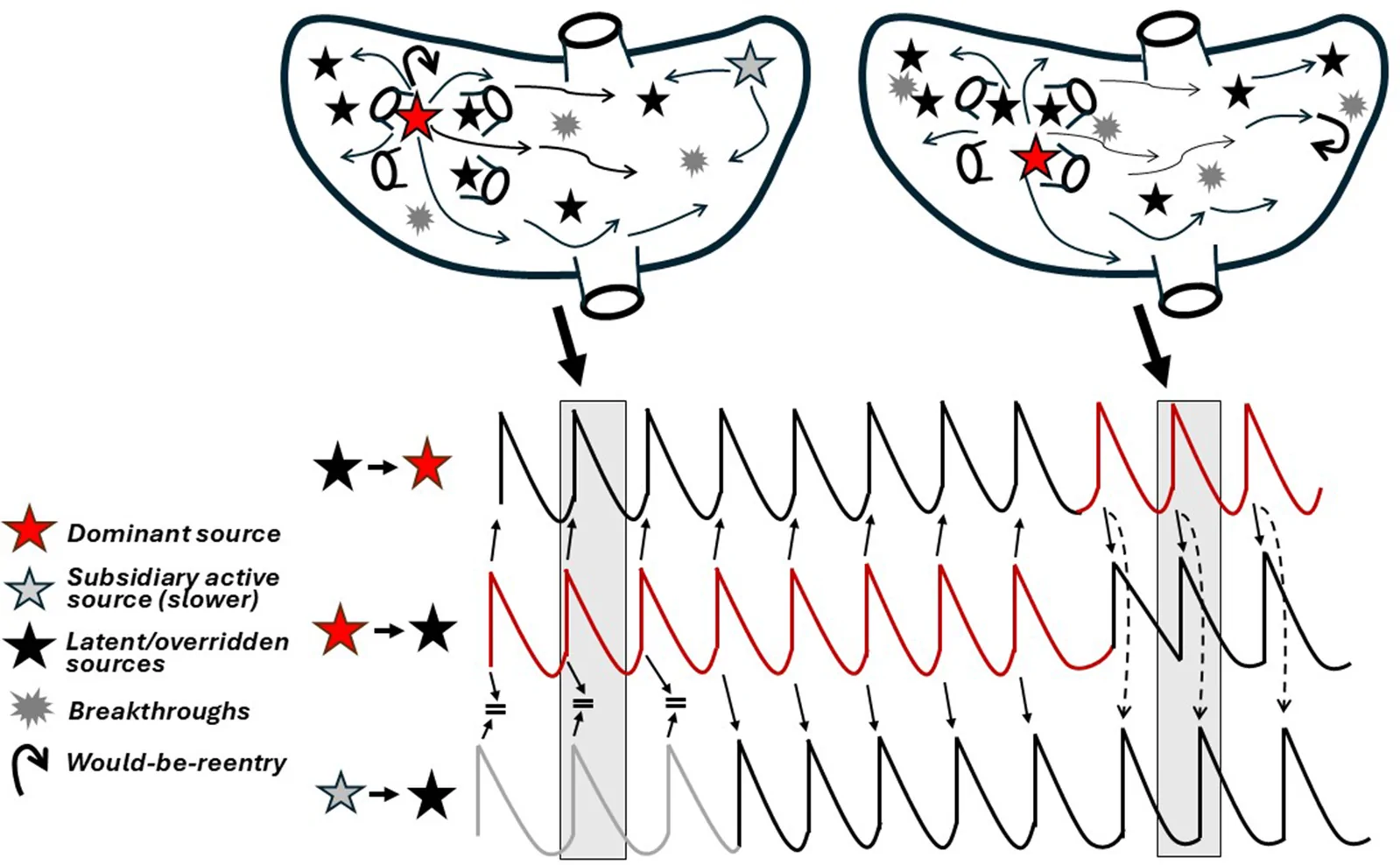
A schematical presentation of the mutual triggering of DAD activity hypothesis. The condition promoting DAD activity (Figure 1) is continuously present during AF throughout the atrium, that may provide a sufficient “arrhythmogenic reserve” for the generation of multiple competitive DAD sources. There should be usually one dominant source at a time (“the currently fastest dominates”) with or without subsidiary active sources. The dominancy site is expected to periodically change. Please see text for details.

It is important to recognize that while there are distinctions between the focal arrhythmic mechanisms (described above), there are significant overlaps as well (115–117). Notably, all focal arrhythmic mechanisms can be dependent on Ca_i_ activity (106, 115, 117, 118). Isoproterenol, known to augment Ca_i_ activity, is used to induce both DAD activity and automaticity *in vitro* and clinical studies (95, 101, 119–121). DAD activity often cannot be reasonably distinguished from automaticity even in isolated tissue slices (100, 101, 117), putting aside clinical studies (121). At present, it is unknown if rapid atrial focal activity recorded during AF is automaticity or triggered activity (or some mixture of these mechanisms). Yet, keeping in mind these uncertainties as well as the above-described *in vitro* vs. *in vivo* focal source discrepancies, among the known focal arrhythmic mechanisms, DAD activity is the best to explain multifocal activity recorded during AF.

Thus, there appears to be a significant “arrhythmogenic reserve” for multifocal DAD activity (or DAD-like activity) during AF capable of maintaining AF (Figure 2).

### Multiple competitive focal sources and dominance

Commonly, there are a substantial spatiotemporal stability during AF (21, 34–40) pointing to a localized dominant driver(s) of AF (21, 40). Spatiotemporal stability recorded during AF can be much easier explained with stationary vs. random sources. It appears that “an average” AF is maintained by a localized dominant driver at a time (21, 40, 41), with or without active subsidiary sources. Such dominance can be broadly explained with multiple competing focal sources during AF. Indeed, repetitive focal sources during AF have various cycle lengths of firing (as reported (6, 7)), and the currently fastest sustained source should naturally act to override all the other sources, covering all, most, or a large part of the atria at a time (the currently “fittest” source dominates). This is a conceivable general scenario.

The *continuity* of sustained focal sources during AF is unknown, largely due to relatively brief recording periods (most commonly ≤ 32 s) (6, 7, 12, 14, 15). It seems to be possible that the same focal source may uninterruptible fire for minutes, less likely for hours, but unlikely for days-weeks. Available data indicate that sustained atrial focal sources self-terminate and usually slow down before self-termination (86, 101, 117). Rapid focal sources seem to have a limited amount of “charge” to rapidly fire. It appears that putative dominant focal sources maintaining AF eventually self-terminate or significantly decelerate, which may trigger latent sources or “clear the road” for subsidiary active sources. Also, the rate of a subsidiary focal source may temporarily exceed that of the dominant source so that this subsidiary source may become the dominant source. Note sustained focal sources during AF may not only have significant inter-source variations in their cycle lengths (6, 7), but also individual focal sources may have significant temporal variabilities in their cycle length, accelerating and decelerating within seconds (6, 86). In Lee et al. studies (6, 7), each of the 8 sustained focal sources detected during AF (in 12 patients) displayed a considerable temporal variability in cycle length within a 32 s recording period (for example, ranging from 130 to 154 ms in one of the sources) (7). Intuitively, if focal sources maintain AF, there should be periodical changes in the dominance site due to an anticipated dynamic competition between the focal sources (“the currently fittest dominates”). Changes in dominance site may not significantly alter general repetitive pattens of activation if different dominant sources are located nearby (Figure 2). Of note, the left atrium (particularly PV regions) is the most probable location of the repetitive focal sources during AF (6, 7, 11, 15, 122), and PV regions readily develop DAD activity/automaticity under conditions of cellular calcium overload (100, 101, 111, 113, 121). The PV regions may be the prime location of the most competitive focal sources during AF.

Thus, AF appears to be maintained by a dominant driver at a time, and such dominancy can be generally explained with the presence of multiple *competitive* focal sources. A periodical switching of dominant sources is expected. AF maintenance can be explained with a reciprocal triggering of competitive sustained DAD sources (or DAD-like sources), with one dominant source at a time (Figure 2).

### Multiple competitive reentries triggering each other?

It is reasonable to assume that multiple competitive reentries triggering and overriding each other may also, like DAD sources, explain the maintenance of AF. It is also possible that competitive focal and reentrant sources may trigger and override each other (in wide range of variations) maintaining AF. Again, reliable 3-D mapping technology is required to test these assumptions. It seems, though, that mutual triggering of reentries is less likely to occur than mutual triggering of focal sources. Mutual triggering of DAD activity is well predictable (i.e., triggered activity by definition) and is based on a massive data set on DAD activity (Figure 1) (94, 95, 108). In contrast, mutual triggering of reentries is poorly understood and is difficult to study (i.e., even a single reentry is either never or rarely detected) (5–12, 14, 15, 23, 26, 27, 61, 62, 66). Also, worth repeating, contemporary AF mapping studies readily record multiple focal activations (including simultaneous) and never or rarely reentries (and only one reentry at a time) (5–12, 14, 15, 23, 26, 27, 61, 62, 66).

### Substrate for AF maintenance

It is generally accepted that there are triggers and substrate of AF, with the triggers commonly being ectopic beat(s) and the substate usually being atrial structural and electrical remodeling causing conduction disturbance and, thus, promoting reentries (2). Yet, there are focal AFs and, thus, along with the reentrant substrate, there is some focal source substrate as well. The latter is poorly understood. In the presence of the reentrant substrate, a single premature beat or a run of rapid activations often induces AF, supporting the “trigger—reentrant substrate” concept. Yet, while the trigger part of this concept is reasonably grounded, the reentrant substrate part is not. Indeed, targeting a wide variety of reentrant substrates (i.e., fibrosis, CFAE, and FIRM) failed to reduce AF occurrence vs. PVI alone in well-conducted RCTs (47–53), and full-circuit reentries during AF are either not recorded at all or recorded infrequently, while focal waves are readily detected (5, 7, 9–15, 28, 31, 32, 61, 62, 65). One may assume that focal source substate may play an important role not only in AF initiation but in AF maintenance as well, along with reentrant substrate.

It is likely that the PV regions have the most arrhythmogenic focal source substrate in the atrium [at least PV regions are the prime source of focal triggers (44)]. PV regions are also capable of sustaining AF (4, 45), presumably by generating both focal and reentrant sources. In non-PV atrial regions, as in PV regions, along with reentrant substrate, there is some focal source substrate as well [focal activations during AF are recorded throughout both atria (5–15)]. While each of these arrhythmogenic substrates can sustain AF, there may be interactions between focal and reentrant substrates throughout the atrium to maintain AF (this matter is practically unknown). It is possible also that in many AF cases the mechanism of initiation and maintenance of AF (i.e., “triggers” and “substrate/driver”, respectively) may be the same arrhythmogenic focal source machinery, with the “trigger” and “driver” being the same or different focal sources. Within the “reciprocal triggering of competitive focal sources” hypothesis (that addresses only AF maintenance, i.e., only “substrate/driver”), the focal source substrate is Ca_i_ -mediated abnormalities throughout the atrium (that are invariably combined with, and related to, the rapid activation rate). It is tempting to speculate that PV regions are the predominant locations of the most competitive focal sources sustaining AF, with non-PV regions generally having less competitive focal sources. In support of this supposition, PV regions are predominant or common locations of the repetitive focal activations during AF (6, 7, 11, 15, 122), and DAD activity/automaticity readily develop in PV regions under the conditions of Ca_i_ overload (100, 101, 111, 113, 121).

Thus, there are reentrant and focal source substrate in the atrium, and both are capable of AF maintenance. The relative contribution of these substrates to AF maintenance as well as their potential “collaboration” in AF maintenance is practically unknown, and these potential contribution and “collaboration” may vary depending on etiology of AF, stage and types of AF, etc.

### Paroxysmal, persistent, and long-standing AF

The condition for the occurrence of multiple DAD sources (i.e., Ca_i_ abnormalities plus rapid activation) is continuously present in all types of AF. It appears that the proposed hypothesis is generally applicable to paroxysmal, persistent, and long-standing AF. Conceivably, PV regions should be greater involved in the generation of “competitive focal sources” in paroxysmal vs. persistent/chronic AF.

### May “mutual triggering of focal sources” explain the success of PVI and CMP?

The CMP remains the most effective approach against AF (42, 43). The original rationale behind the CMP is that atrial compartmentation reduces the chance of current reentries to continue and novel reentries to appear (42, 43). This rationale is based on the belief that AF is sustained by simultaneous multiple unstable reentries [as it was “established” in 1980th—1990th (42, 57, 59, 60)]. However, contemporary AF mapping studies either do not detect reentry at all or detect a single reentry at a time (5–14), questioning the “multi-reentrant” rationale for the CMP. The anti-AF action of the CMP is likely to be largely due to the PVI (43). The PVI is a proven anti-AF strategy and the prime rationale for this strategy is that the PV regions generate most of the triggers inducing reentries (mostly outside of the PV regions) sustaining AF, and, therefore, the PVI is effective against AF (4, 44, 45). The “reentrant” part of the reasoning, however, is not in line with no or rare detection of reentries in AF mapping studies (5–15) as well as with the failure of reentrant substrate amendments (i.e., fibrosis, CFAE, FIRM, linear lesions, etc.) to reduce AF occurrence in the RCTs (47–52). It is well-recognized that the mechanisms of anti-AF efficacy of the PVI and CMP are multifactorial and complicated (4, 43, 45). Among many factors, partial vagal denervation of PV regions, that may occur secondary to the PVI and CMP, may contribute to anti-AF efficacy of the PVI and CMP (as discussed later). Although there are wide ranges of probabilities and uncertainties, anti-AF success of the PVI and CMP can be generally explained with the interruption of mutual triggering of focal sources located in different atrial areas (seems mostly in the PV regions), and self-termination of active source(s) within the isolated atrial segments (Figure 3).

**Figure 3:**
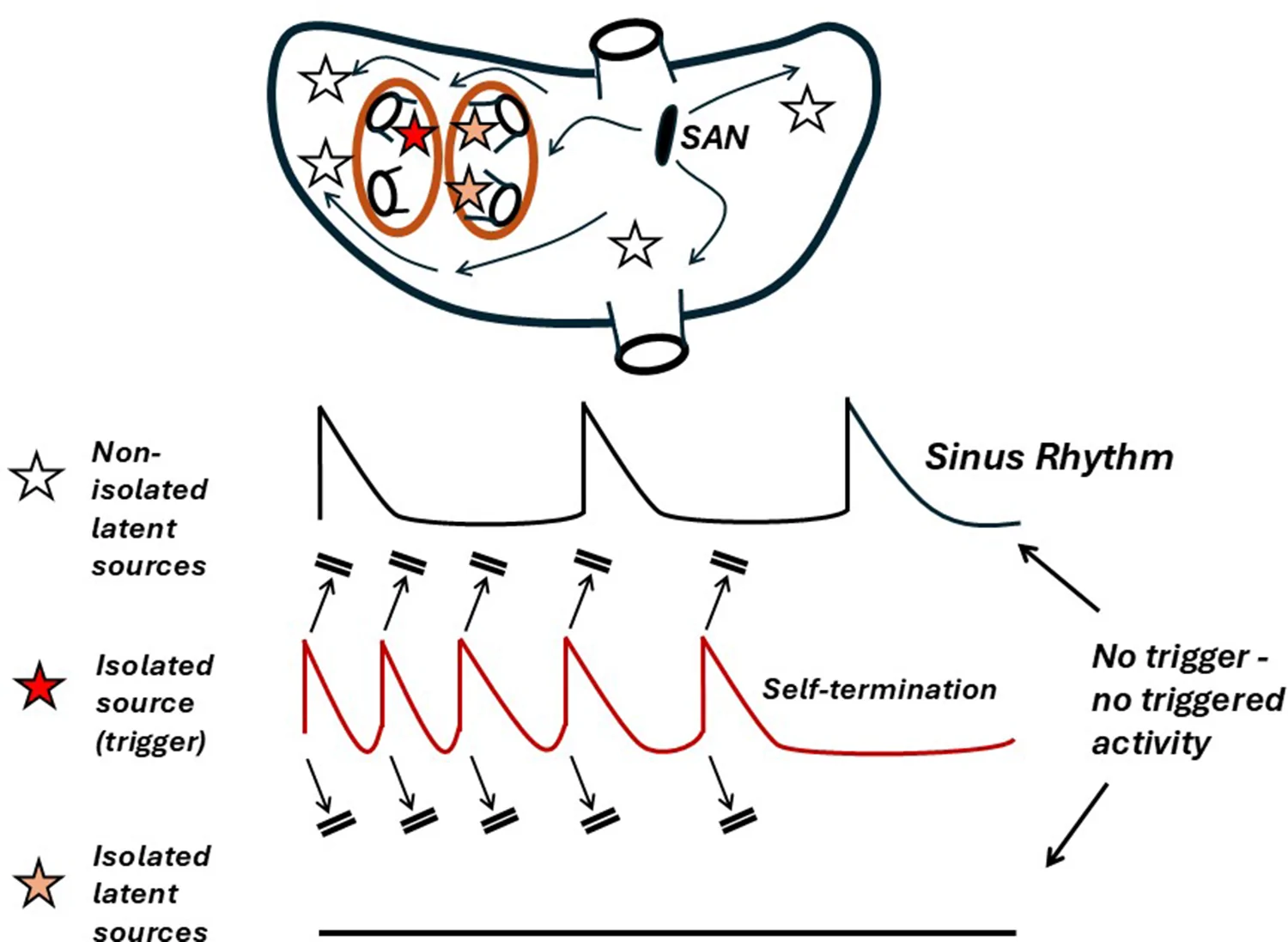
Anti-AF success of PV isolation and Cox-Maze procedure. Due to interruption of mutual triggering of focal sources? For illustrative purposes, only PV isolation is shown (PV isolation seems most critical for anti-AF success of the Cox-Maze procedure). Isolation of PV regions should reduce the chances of mutual triggering of multiple DAD sources, thus, reducing the chances of AF maintenance and re-initiation of AF. Latent DAD sources need rapid activation to develop triggered activity (i.e., “no trigger—no triggered activity”). SAN—sinoatrial node.

### Autonomic influences and AF maintenance

Autonomic nervous system (ANS) may significantly affect AF generation (123, 124). This topic, however, remains poorly understood and controversial (123). Notably, in 2004 Pappone at al (124). demonstrated that vagal autonomic denervation of PV regions significantly contributed to the reduction of AF occurrence in addition to the PVI. Later, many studies (123, 125–127) [but not all (128)] targeting ganglionated plexi have generally supported the “vagal denervation may reduce AF” idea. Arrhythmic mechanism of vagally-mediated promotion of AF and anti-AF mechanism of vagal autonomic denervation appear to be critically related to atrial effective refractory period (ERP). Vagal tone shortens atrial ERP (known to promote AF) and vagal denervation prolongs atrial ERP (129, 130), explaining anti-AF effect of such denervation. Parasympathetic impulses, along with atrial ERP shortening, may also increase atrial electrical heterogeneities, both these alterations act to promote reentries (131). However, many high-quality mapping studies did not detect reentries but readily recorded focal activation patterns on atrial surfaces during vagally-mediated AF (55, 63, 132). There is reasonable evidence that vagally-mediated AF can be sustained by focal sources (63, 132–134). It appears that both reentrant and focal arrhythmic mechanisms can sustain vagally-mediated AF.

The mechanism of vagally-induced rapid focal sources is unknown. Generally, vagal tone acts to suppress focal source activity (at normal and slow activation rates). However, in the setting of rapid activation (producing Ca_i_ loading/abnormalities), vagal influences alone (106, 107) and in combination with adrenergic influences (135–137) may cause/promote the appearance of arrhythmic focal sources (mostly late-phase 3 EAD activity) in the atrium [particularly in PVs (135–137)]. The cellular mechanism of “conventional” DAD and late phase 3 EAD is similar, i.e., elevation of Ca_i_ causing activation of the inward Na/Ca exchange current (106, 107, 136). A critical difference is that conventional DAD is caused by spontaneous SR calcium (94, 95, 108) and the late phase 3 EAD by normal SR calcium release on the background of a significant abbreviation of atrial repolarization (106, 107). The “classical” late phase 3 EAD activity develops following a period of rapid rate and pause, with the first spontaneous action potential developing at slightly depolarized RMP (thus, late phase 3 EAD), but the following action potentials commonly switching to normal near/normal RMP (106, 135–137) (i.e., these action potentials look like DAD activity). The late phase 3 EAD activity has been thought to initiate but not maintain AF (106, 107, 136, 137).

Principally, vagally-induced shortening of atrial repolarization and Ca_i_ loading is critical for the development of late phase EAD activity (106, 107, 135–137). Notably, there is a substantially greater level of diastolic Ca_i_ in atrial cells isolated from AF vs. sinus rhythm patients (as measured at normal pacing rates) (110), and rapid rate is expected to significantly increase Ca_i_ loading/abnormalities. Also, AF significantly decreases Ca_i_ transient amplitude but appears to cause proportionally less shortening of the duration of Ca_i_ transient than repolarization (110). Ca_i_ transient may significantly extend beyond 100% repolarization in atrial cells isolated from patients with persistent AF (110). This should promote the focal activity induced by “normal” SR release during AF. It appears that during AF atrial repolarization shortening induced by vagal tone and by the other influences may promote non-conventional DAD activity secondary to elevated Ca_i_ during the diastolic interval (such elevated Ca_i_ can be due to the combination of normal SR release and a “steady” increase of diastolic Ca_i_; Figure 4).

**Figure 4:**
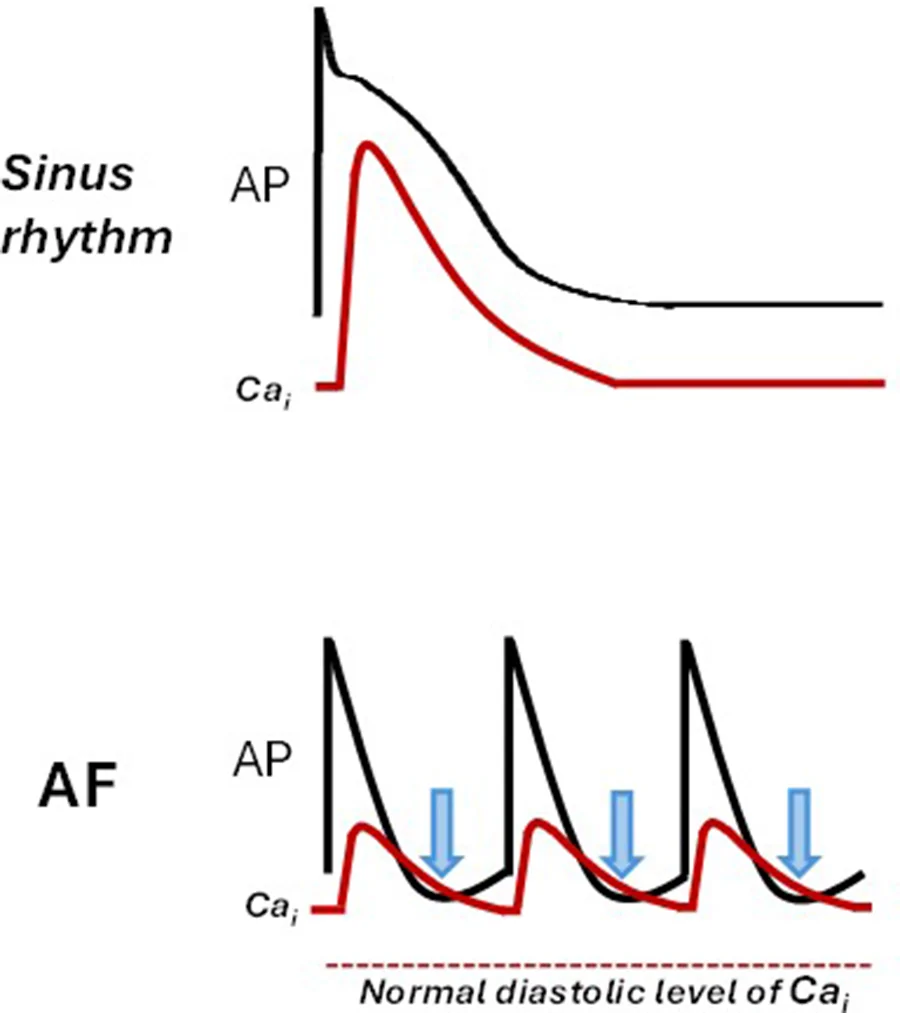
Schematical presentation of hypothetical non-conventional DAD activity during AF. Atrial repolarization shortening (due to vagal and other influences) should promote non-conventional DAD activity induced by the elevated Cai during diastolic interval (such elevation is due to the combination of normal SR calcium release and “permanently” increased Cai during AF). Blue arrows indicate the timing of the inward Na/Ca exchange current, causing the initial “kick” for the DAD activity. Please see the text for details.

Theoretically, during AF the “steady” elevation of diastolic Ca_i_ may be more important than normal SR release or even sufficient for the development of non-conventional DAD activity **(**Figure 4). SR calcium release during AF is poorly defined but is known to be decreased (110, 138). One may assume that there is a substantial dynamic variability in the amount of “normal” SR calcium release during AF (ranging from relatively significant to no release). Theoretically, the proposed non-conventional DAD activity may contribute to AF maintenance, along with the other arrhythmic mechanisms (particularly reentry and conventional DAD activity). This non-conventional DAD activity is a “raw” assumption. Yet, this assumption appears to be worth consideration and investigation.

Importantly, considering that AF promotes spontaneous SR calcium leak (109, 110, 112), DAD activity during AF may encompasses the conventional and non-conventional DAD activities, with each of these DAD subtypes potentially operating individually or/and in various mixed combinations. Also critical, both parasympathetic and sympathetic tones are permanently present during AF in the human (in various proportions) and a combined activation of them may importantly contribute to DAD generation during AF. Of note, triggered activity in PVs most readily occurs with the combination of vagal and adrenergic stimulations (135–137). Sympathetic influence-induced elevation of Ca_i_ causes/promotes conventional DAD activity (94, 95, 108) and it should cause/promote non-conventional DAD activity as well. Knowing that ANS affect atrial repolarization and Ca_i_, one may assume that ANS activity may significantly modify the proposed “reciprocal triggering of competitive focal sources” via affecting the appearance, disappearance, acceleration, and slowing of focal sources during AF. Also, the PVI and CMP may reduce ANS influences on the PV regions, and such influences may partially account for the anti-AF effect of these approaches.

Thus, the ANS may significantly influence AF maintenance, with vagally-induced shortening of atrial repolarization being the most probable and substantial pro-AF effect of the ANS. Vagally-mediated abbreviation of atrial ERP may promote reentry but, conceivably, may promote Ca_i_ -dependent triggered activity as well.

### “Pharmacological” analysis of AF mechanism

Electrophysiological alterations induced by anti-AF agents before AF cardioversions appears to be consistent with a termination of a focal but not reentrant mechanism (3, 92). Notably, drug-induced AF termination is associated with prolongation of the temporal excitable gap (139). A focal source termination is naturally associated with prolongation of the temporal excitable gap (3). In contrast, most available data and theories indicate that anti-AF agents terminate reentry with elimination of the excitable gap (140–143). Moreover, I_Kr_ blockers shorten the excitable gap in all types of reentries (141, 144–147). For details, please see (3, 92).

### Reentry and AF maintenance

It is well accepted that reentry can maintain AF (2, 58, 59). However, the dominant role of reentry in the maintenance of AF, thought well established 1–3 decades ago, has not been proven and is debatable now (1, 3, 8). It appears that the role of reentry in AF maintenance has been exaggerated and that of focal sources overlooked. This manuscript argues that focal sources are more likely to be the primary driver of AF than reentry.

### What is the dominant mechanism of AF maintenance?

There are four general concepts of AF maintenance 1) “random wavelets”, “multiple random reentries”, “mother reentry”, and “focal source(s)”. The “reciprocal triggering of competitive focal sources” should be considered as an upgrade of the general focal source theory. The “random wavelets” hypothesis does not seem to be robust. This theory is inconsistent with 1) spatiotemporal periodicity commonly recorded during AF (21, 34–41), 2) distinct and repetitive F waves often documented during AF) (148, 149) (such F waves can be readily explained with single/dominant source but not with multiple random wavelets), and 3) a common presence of some AF cycle length (148, 149) (hundreds/dozens of simultaneous random wavelets should not produce AF cycle length). Also, the fact that a stable remote rapid source can readily produce fibrillatory wavelets (58, 78) indicates that random wavelets during AF are most likely the consequence of rapid activation and not the cause of it.

The evidence for focal and reentrant sources maintaining AF is relative, questionable, non-conclusive, and contradictive, as discussed previously in the manuscript. Table 1 presents relatively reliable data supporting and arguing against reentrant and focal mechanisms of AF maintenance. These mechanisms do not exclude each other. AF can be maintained by various AF mechanisms (1, 2, 4, 45). The newly introduced “reciprocal triggering of competitive focal sources” is a potential mechanism that may sustain AF.

**Table 1:** What is the dominant mechanim of AF maintenance?

Multiple random reentries	
Consistent	Inconsistent
Anti-AF success of Cox-Maze procedure	Contemporary AF mapping studies detect either a single reentry at a time or no reentry at all
	Spatiotemporal periodicity

“Mother” reentry	
Consistent	Inconsistent
Spatiotemporal periodicity	The vast majority of AF mapping studies never detect sustained^a^ reentries, with many studies never detecting any reentry.
	Failure of reentrant substrate modification to reduce AF occurrence in the RCTs.

Reciprocal triggering of competitive focal sources	
Consistent	Inconsistent
Multifocal activation patterns are regularly detected during AF	Significant number of AF mapping studies never find sustained^b^ focal activations
Spatiotemporal periodicity	
Anti-AF success of Cox-Maze and PVI procedures	

a For more than several seconds.

b For more than several seconds. Spatiotemporal periodicity is caused by a sustained source(s).

## Conclusion

Reciprocal triggering of competitive DAD sources, with one dominant source at a time, may be the primary mechanism of AF maintenance, broadly explaining the spatiotemporal stability during AF as well as anti-AF success of Cox-Maze and PV isolation procedures.
